# Characteristics and phylogenetic analysis of the complete chloroplast genome of *Rubus quinquefoliolatus* T.T.Yu & L.T.Lu (family Rosaceae)

**DOI:** 10.1080/23802359.2022.2098859

**Published:** 2022-07-25

**Authors:** Ke Zhang, Yongfei Yin, Shanyong Yi, Haitao Li

**Affiliations:** a Department of Pharmacy, Anhui University of Chinese Medicine, Hefei, PR China; b Department of Biological and Pharmaceutical Engineering, West Anhui University, Luʼan, PR China; c Anhui Engineering Laboratory for Conservation and Sustainable Utilization of Traditional Chinese Medicine Resources, West Anhui University, Luʼan, PR China; d Yunnan Branch Institute of Medicinal Plant, Chinese Academy of Medical Sciences and Peking Union Medical College, Jinghong, PR China

**Keywords:** *Rubus quinquefoliolatus*, complete chloroplast genome, phylogenetic analysis

## Abstract

*Rubus quinquefoliolatus* T.T.Yu & L.T.Lu is a climbing shrub belonging to the Rosaceae family. It is widely distributed in the provinces of Yunnan and Guizhou in China. In this study, we sequenced the first complete chloroplast genome (cpDNA) sequence of *R. quinquefoliolatus*. The results showed a genome length of 156,489 bp, which is composed of a large single-copy (LSC) of 86,103 bp, small single-copy (SSC) of 18,844 bp, and two inverted repeats of 25,771 bp each. The whole chloroplast genome encodes 131 genes, including 86 coding sequences, 37 tRNAs, and eight rRNAs. Phylogenetic analysis revealed that *R. quinquefoliolatus* is closely related to *R. lineatus* and *R. pentagonus*.

*Rubus quinquefoliolatus* T.T.Yu & L.T.Lu (1982) is a perennial climbing shrub of the *Rubus* genus of the Rosaceae family that is widely distributed, and native to the provinces of Yunnan and Guizhou in China (Yu and Lu 1982). The plant is used as herbal medicine and its fruit as food (Si et al. 2015). It can reach 1.5 m in height, and its branches are cylindrical, ranging from grayish brown to purple-brown. They are pilose when young, fall off when old, and have few prickles. The leaves are often palmate, with five leaflets that are elliptic lanceolate or rhombic lanceolate. *R. quinquefoliolatus*, and other plants of the same genus, are usually considered to have a group with high economic, medicinal, and ecological values (Duan et al. 2006). They are an important resource for small berry fruit trees, which have the high nutritional value and many biological functions, such as anti-oxidant, anti-bacterial, hypoglycemic, and cardiovascular protection (Han and Liu 2009). This genus is one of the most challenging genera in the study of flowering plant phylogeny (Sochor et al. 2015), and it is also a good material for studying plant reproductive evolution. Here, the complete chloroplast genome (cpDNA) of wild *R. quinquefoliolatus* was characterized, which can provide useful informatics data for the phylogeny of *Rubus* genus and further enrich their evolutionary research.

Fresh leaves of *R. quinquefoliolatus* were collected from Laojun Mountain, Dulong Town, Maguan County, Yunnan Province, China (22°56′ N, 104°33′ E). The voucher specimen was deposited in the Herbarium of the Anhui University of Chinese Medicine (accession number: WYXGZ-2015001, Yongfei Yin, yinyongfei@ahtcm.edu.cn). In China, *R. quinquefoliolatus* is not a protected plant; hence, we did not collect it from private or protected areas that require permission. Total genomic DNA was obtained according to a modified CTAB protocol (Doyle and Doyle 1987). Genome sequencing was performed using the Illumina HiSeq platform at Hefei Biodata Biotechnologies, Inc. (Hefei, China). The cpDNA sequences were assembled using SPAdes assembler 3.10.0 (Bankevich et al. 2012). GeSeq (Tillich et al. 2017) was used to annotate all genes under the default parameters to predict protein-coding, rRNA, and tRNA genes.

The cpDNA of *R. quinquefoliolatus* was deposited in NCBI under the accession number OM691693 with a genome size of 156,489 bp. The genome consists of four regions, namely: large single-copy (LSC), small single-copy (SSC), and two inverted repeats (IRs). Specifically, LSS and SCC contained 86,103 and 18,844 bases, respectively, while IRa and IRb each contained 25,771 bp. In total, 131 genes, comprising of 86 coding sequences, 37 tRNAs, and eight rRNAs, were predicted. Among them, 18 contained two exons, and four genes (*paf*I, two *rps*12, and *clp*P1) contained three exons. Seven protein-coding genes, five tRNAs, and four rRNA genes were duplicated in the IR regions of the *R. quinquefoliolatus* chloroplast genome.

To compare evolutionary differences, a phylogenetic relationship was constructed based on the complete cpDNA sequence of *R. quinquefoliolatus* and its same genus species. In detail, 61 cpDNAs of the genus *Rubus*, including *R. quinquefoliolatus*, and two out-groups (*Malus doumeri* and *Prunus domestica*), were used for the alignment using MAFFT v7.307 (Katoh and Standley 2013). The phylogenetic tree was constructed via the maximum-likelihood method using the FastTree version 2.1.10 (Price 2010) under the best-fit nucleotide substitution model with GTR + γ, Shimodaira–Hasegawa test. Phylogenetic analysis indicated that *R. quinquefoliolatus* is closely related to *R. lineatus* and *R. pentagonus* (Figure 1). The cpDNA sequence of *R. quinquefoliolatus* lays a vital foundation for phylogenetic and evolutionary studies of *Rubus* species.

**Figure 1.**
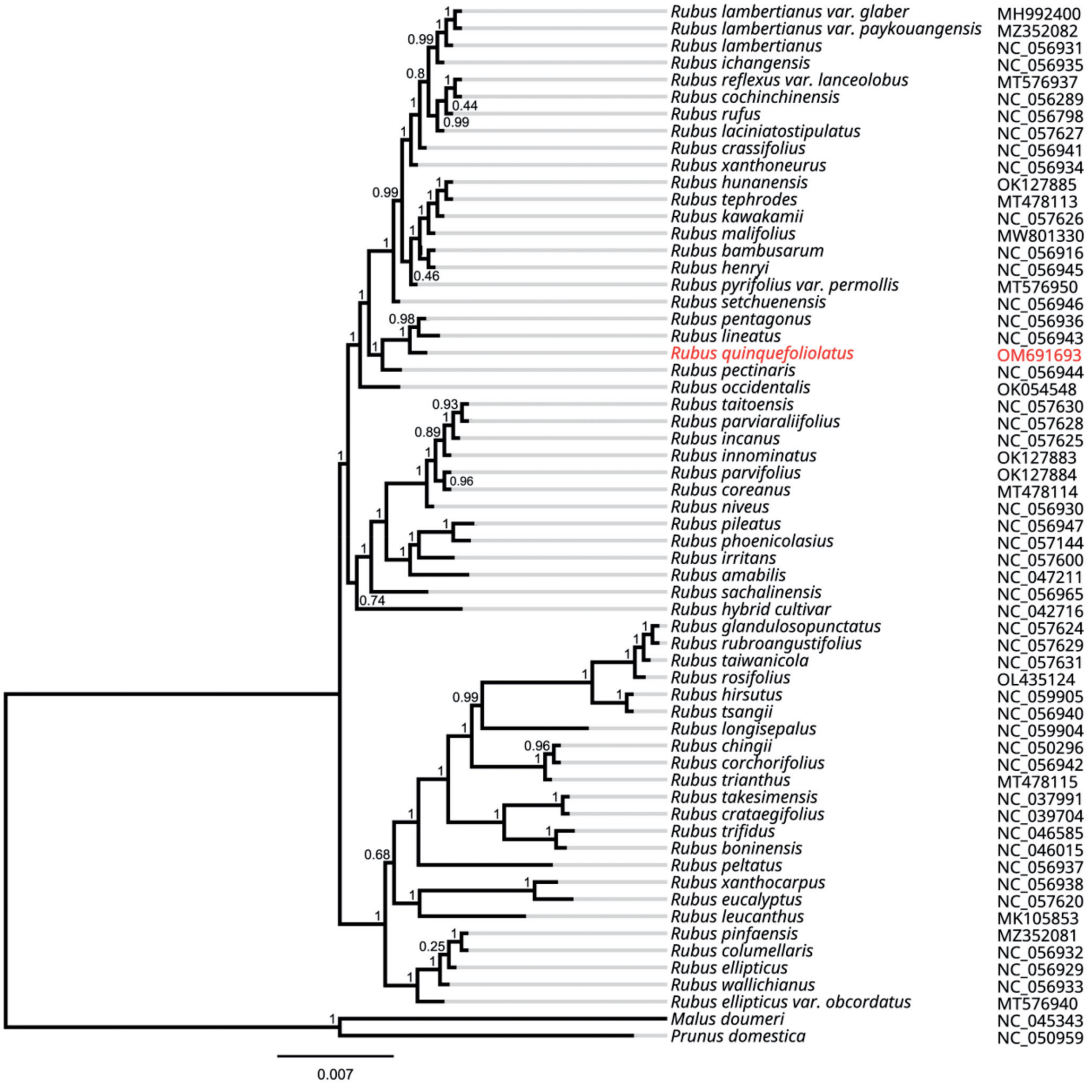
Phylogenetic tree inferred by maximum-likelihood (ML) method based on 61 representative species. *Malus doumeri* and *Prunus domestica* were used as outgroup. A total of 1000 bootstrap replicates were computed and the bootstrap support values are shown at the branches. Accession numbers are shown in the figure.

## Data Availability

The genome sequence data of *R. quinquefoliolatus* that support the findings of this study are openly available in GenBank of NCBI at https://www.ncbi.nlm.nih.gov/ under the accession no. OM691693. The associated BioProject, SRA, and Bio-Sample numbers are PRJNA803817, SRR17913405, and SAMN25688534, respectively.
